# Supplementary material for: Phagosomal signalling of the C-type lectin receptor Dectin-1 is terminated by intramembrane proteolysis
Source: Nat Commun. 2022 Apr 6;13:1880. doi: 10.1038/s41467-022-29474-3 (PMC8987071; doi:10.1038/s41467-022-29474-3)
Supplement: Supplementary file 1 — Supplementary Information [file 41467_2022_29474_MOESM1_ESM.pdf]

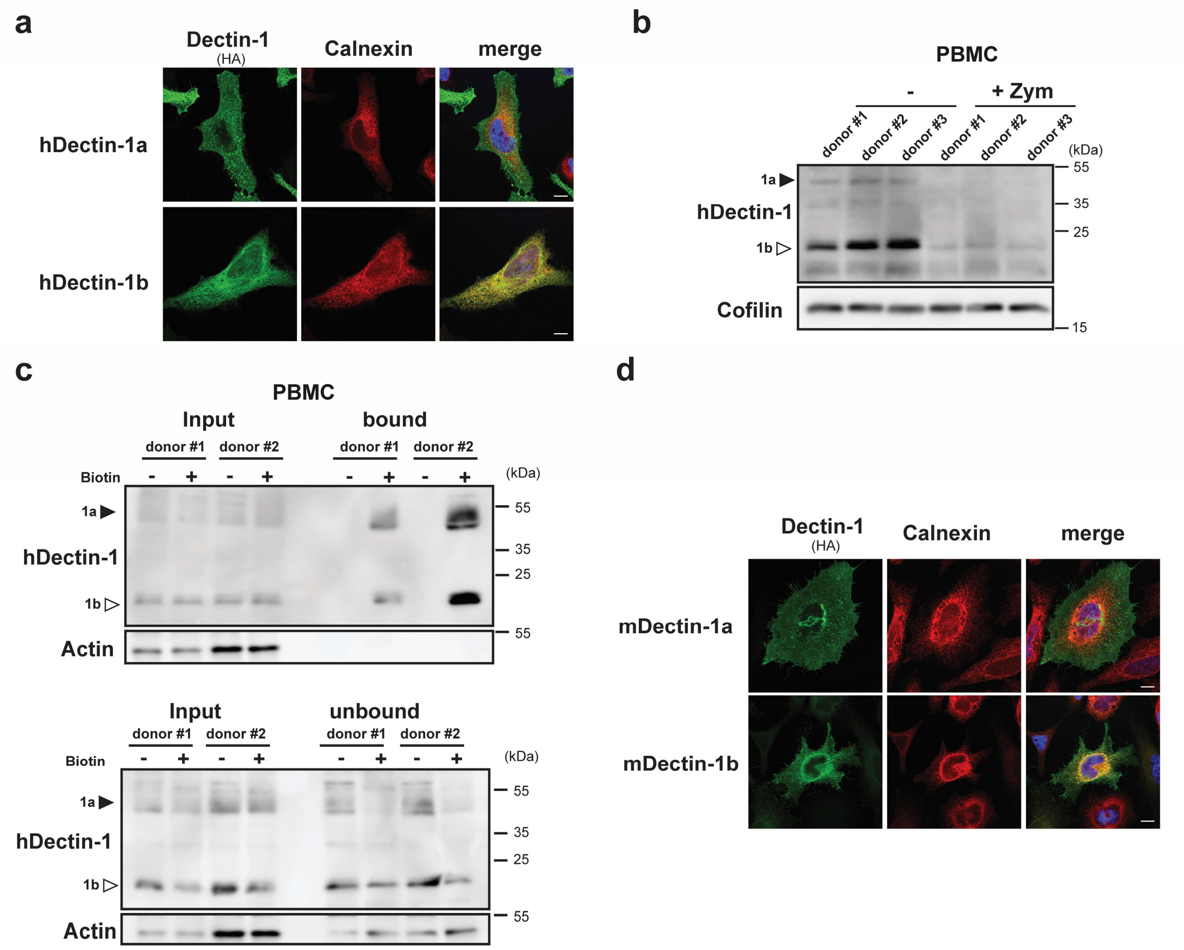

SUPPL. FIGURE 1. Subcellular localisation of the human and murine Dectin-1a and Dectin-1b isoforms. a HeLa cells were transfected with either HA-hDectin-1a or HA-hDectin-1b fixed with 4% PFA and Dectin-1 was stained using anti-HA. The endoplasmic reticulum was visualised using anti-Calnexin. Scale bar, 10 µm. N=3, n=3. **b** Human PBMCs were treated for 4 h with 100 µg/ml Zymosan (Zym) and subsequently analysed for Dectin-1 levels by Western Blotting. N=3, n=9. **c** Surface proteins of human PBMCs were labelled with a membrane-impermeable biotinylation reagent and subsequently precipitated from cell lysates using streptavidin-beads. Bead eluates (bound), lysates (Input) and unbound fractions were analysed by Western Blotting for presence of Dectin-1. Bands representing Dectin-1a and Dectin1-b were labelled with closed and open arrowheads, respectively. N=3, n=6. **d** The experiment described in a) was repeated employing HA-mDectin-1a or HA-mDectin-1b. Scale bar, 10 µm. N=3, n=3.

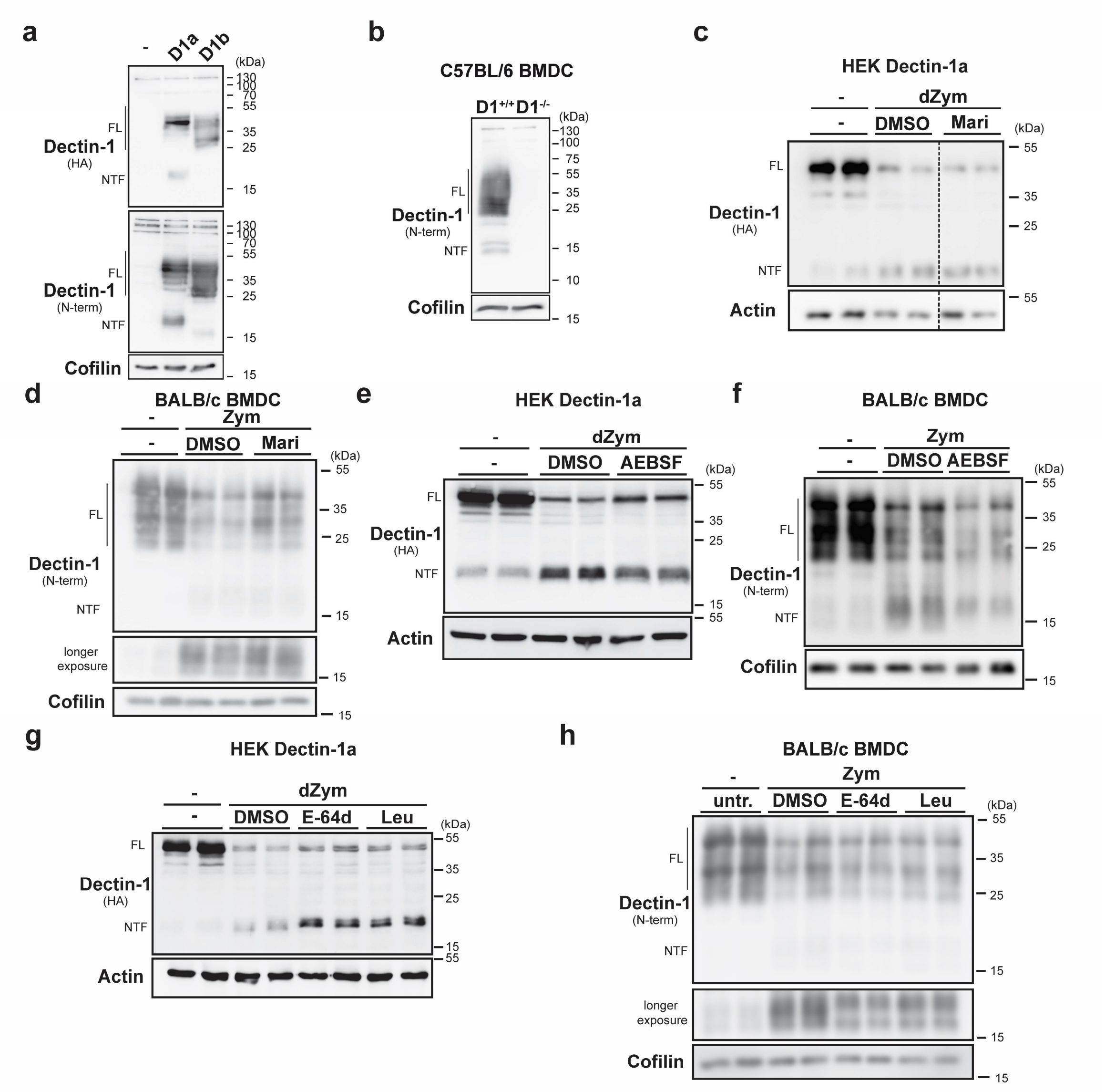

SUPPL. FIGURE 2. Characterisation of Dectin-1a processing using different protease inhibitors. a HEK cells were transiently transfected with either an empty vector (-) or HA-mDectin-1a-FLAG or HA-mDectin-1b-FLAG prior to lysis. Blots were detected using either anti-HA or the newly generated Dectin-1 antibody targeting the N-terminus of the murine receptor. Throughout the Figure, full length (FL) Dectin-1 as well as the corresponding N-terminal fragment (NTF) are highlighted. N=2, n=2. **b** Specificity of the newly generated Dectin-1 antibody was validated in BMDC derived from either wild type ($D1^{+/+}$) or Dectin-1-deficient *Clec7a*$^{-/-}$ mice ($D1^{-/-}$) on a C57BL/6 background by Western Blotting. N=1, n=1. **c** HEK cells stably overexpressing HA-mDectin-1a-FLAG were incubated for 30 min with 10 µM Marimastat (Mari) prior to stimulation with 50 µg/ml depleted Zymosan (dZym) for 6 h. Proteolysis of Dectin-1 was analysed by Western Blotting. N=2, n=4. **d** The same experiment was conducted employing wild type BALB/c BMDC which were treated with 100 µg/ml Zymosan (Zym) instead of dZym. N=3, n=6. **e** HEK cells overexpressing HA-mDectin-1a were incubated with 500 µM AEBSF for 30 min prior to stimulation with 50 µg/ml dZym. Samples were analysed by Western Blotting for Dectin-1 proteolysis. N=3, n=6. **f** Same set-up as described in e, but employing BALB/c BMDC which were treated with 100 µg/ml Zym instead of dZym. N=3, n=6. **g** HEK cells overexpressing HA-mDectin-1a-FLAG were stimulated with 50 µg/ml dZym for 6 h after pre-incubation with 40 µM E64-d or 2.5 µM Leupeptin (Leu). E64-d: N=4, n=8; Leu: N=3, n=6. **h** Wild type BALB/c BMDC were treated as described in f) but with 100 µg/ml Zym instead of dZym. N=3, n=6.

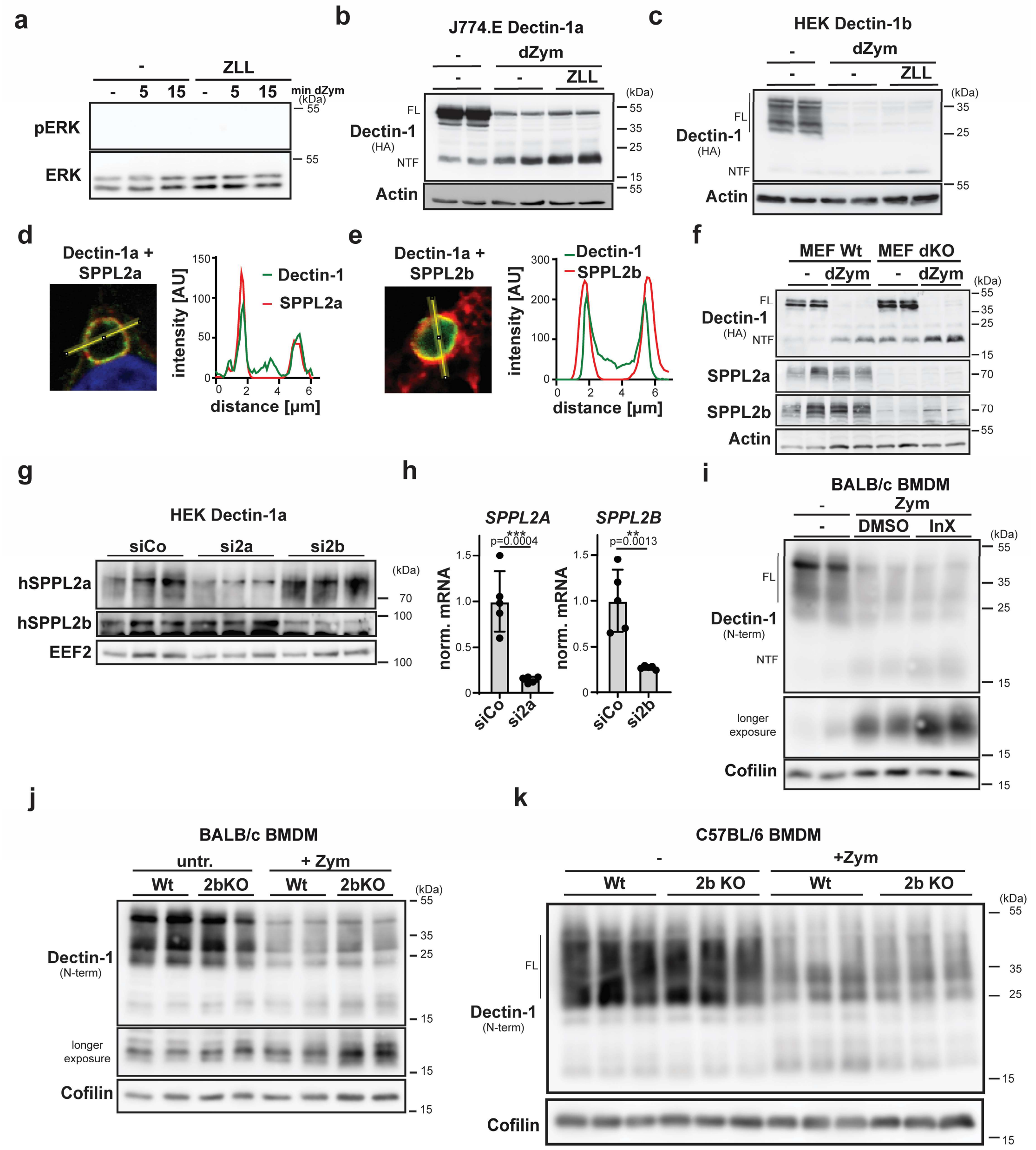

SUPPL. FIGURE 3: Proteolysis of the Dectin-1a N-terminal fragment depends on the intramembrane proteases SPPL2a/b. a HEK cells stably transfected with an empty vector were serum-deprived for 1 h and then stimulated with 50 µg/ml depleted Zymosan (dZym) for the indicated time points. The phosphorylation status of ERK1/2 was analysed by Western Blotting applying specific antibodies. N=2, n=2. **b** J774.E cells stably overexpressing HA-mDectin-1a-FLAG were pre-treated for 30 min with 40 µM ZLL and then stimulated for 6 h with 50 µg/ml depleted Zymosan (dZym) as indicated. After lysis, Dectin-1 processing was visualised by Western Blotting. Throughout the Figure, full length (FL) Dectin-1 as well as the corresponding N-terminal fragment (NTF) are highlighted. N=2, n=4. **c** HEK cells stably overexpressing HA-mDectin-1b-FLAG were treated as described in b). N=2, n=4. **d** Magnification of representative phagosomes of HeLa cells co-expressing HA-mDectin-1a (green) and mSPPL2a-myc (red) and stimulated with 50 µg/ml dZym for 1 h as shown in Figure 3. Fluorescence intensity plots were generated using ImageJ software. N=2, n=2. **e** Same as in d) but cells were co-transfected with HA-mDectin-1a (green) and mSPPL2b-myc (red) as depicted in Fig. 3e. N=2, n=2. **f** Either wild type (Wt) or SPPL2a/b-double deficient MEFs (dKO) stably transfected with HA-mDectin-1a-FLAG were either left untreated (-) or

stimulated for 6 h with 50 µg/ml dZym and subjected to Western Blot analysis employing the indicated antibodies. N=4, n=5. **g** Reduced protein levels of SPPL2a and SPPL2b upon transfection of HA-mDectin-1a-FLAG expressing HEK cells with control or *SPPLA/B*-targeting siRNA was validated by Western blotting. N=2, n=5. **h** siRNA-mediated knockdown of *SPPL2A* and *SPPL2B* in stably HA-mDectin-1a-FLAG expressing HEK cells (Figure 3i) was validated by qPCR employing primers against *SPPL2A*, *SPPL2b* and *TUBA1C* as housekeeping gene. N=5, n=5. Two-tailed unpaired Student's t-test. Bars depict Mean±SD. ** $p \leq 0.01$; *** $p \leq 0.001$. **i** Wild type BALB/c BMDM were treated for 30 min with 1 µM inhibitor X prior to stimulation with 100 µg/ml Zym for 6 h and Western Blot analysis. **j** BMDM from either wild type or SPPL2b-deficient mice were treated for 6 h with 100 µg/ml Zym. Dectin-1 levels were finally analysed by Western blotting. N=2, n=6. **k** The same experiment as described in j was repeated with mice with the same genotypes but on C57BL/6 background. N=2, n=6.

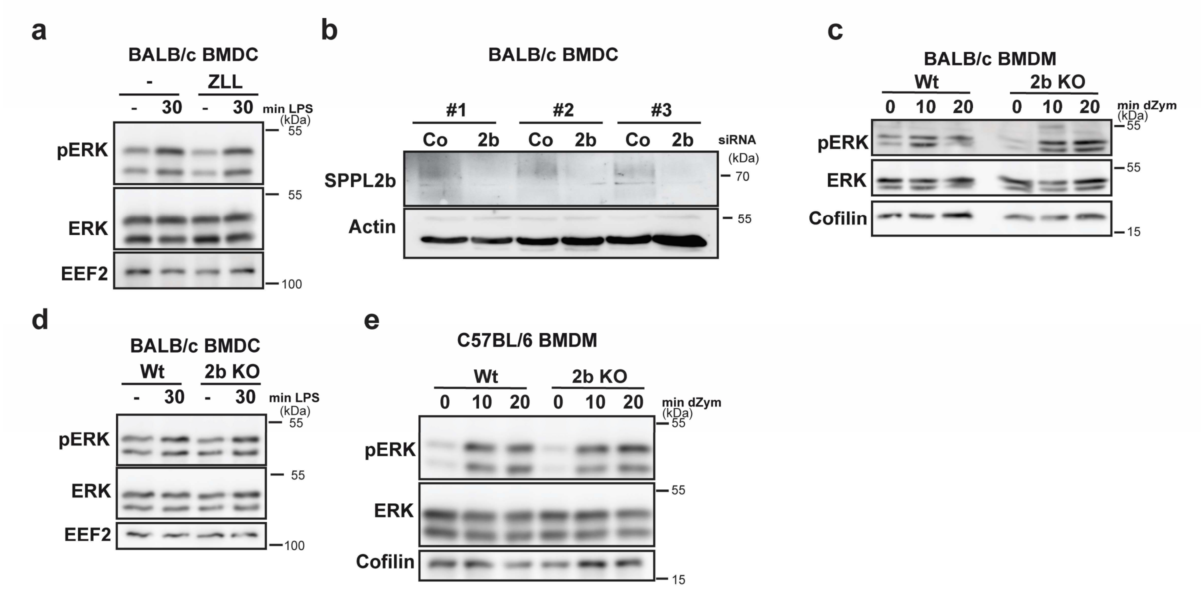

SUPPL. FIGURE 4: The intramembrane protease SPPL2b influences signalling of Dectin-1a, but not of Dectin-1b or TLR4. a BMDCs from BALB/c wild type mice were serum-starved for 1 h in the presence of either DMSO (-) or 40 µM ZLL to inhibit SPPL2 proteases. Subsequently, cells were stimulated for 30 min with 500 ng/ml LPS and analysed for phosphorylated as well as total ERK1/2 by Western blotting. N=3, n=7. **b** Knockdown of SPPL2b in BALB/c wild type BMDC upon treatment with specific siRNAs was validated by Western Blotting using an antibody targeting the C-terminus of SPPL2b. N=1, n=3. **c** Either wild type (Wt) or SPPL2b-deficient (2b KO) BALB/c BMDM were serum-deprived for 1 h and then stimulated with 50 µg/ml dZym for the indicated time points. MAPK phosphorylation was assessed by Western Blotting. N=2, n=5. **d** BMDC from either Wt or 2b KO BALB/c mice were starved for 1 h prior to incubation with 500 ng/ml LPS for 30 min. Phosphorylation of ERK was monitored by Western blotting. N=2, n=5. **e** Wt or 2b KO C75BL/6 BMDM were incubated for 1 h in serum-depleted medium prior to stimulation with 50 µg/ml dZym for the indicated time points. Phosphorylation of ERK was subsequently analysed by Western blotting. N=2, n=6.

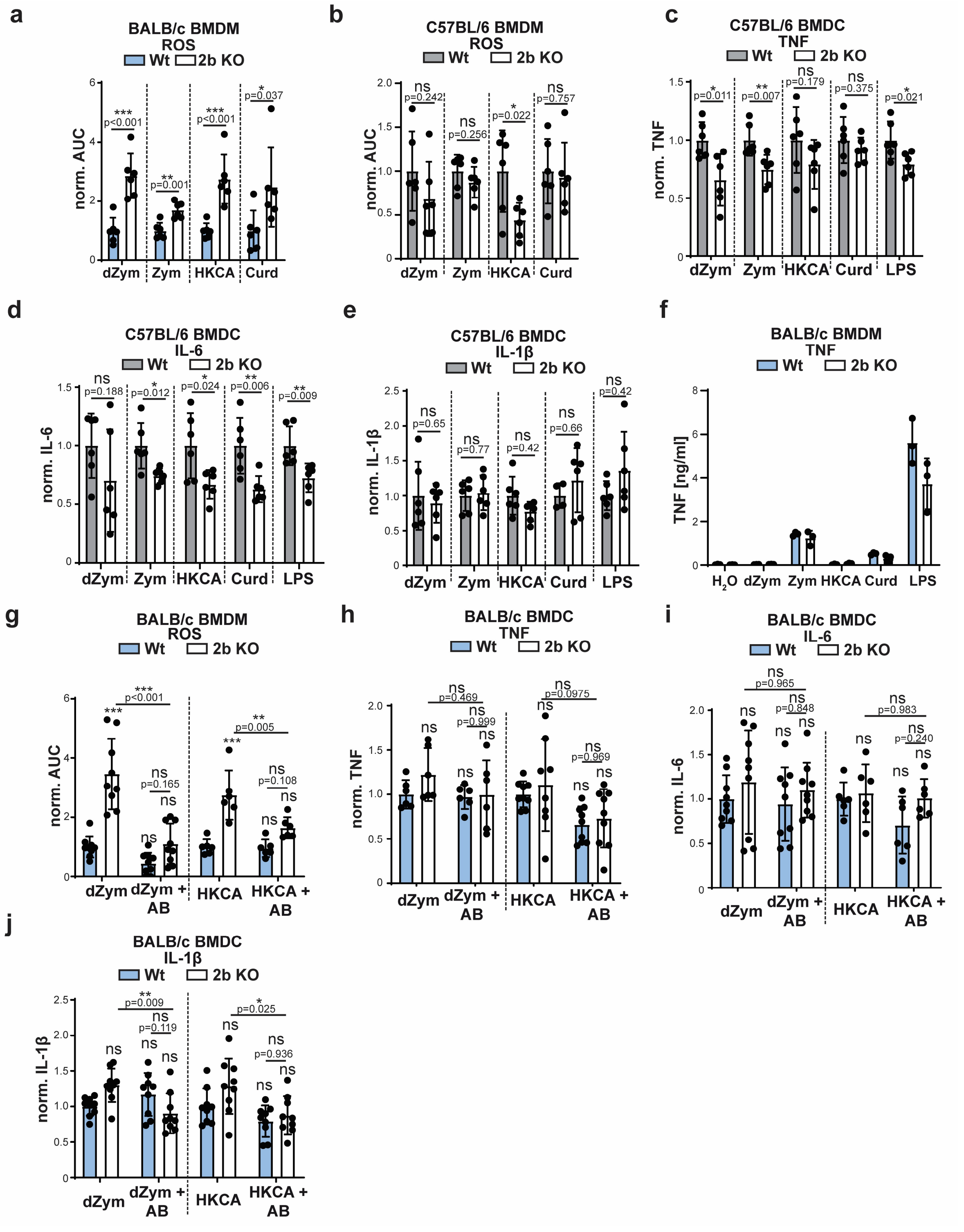

SUPPL. FIGURE 5: Impact of SPPL2b deficiency on anti-fungal ROS and cytokine responses of macrophages and dendritic cells. a BALB/c BMDM from wild type (Wt) or SPPL2b-deficient (2b KO) mice were treated with either 50 µg/ml dZym, 50 µg/ml Zym, MOI 10 HKCA or 200 µg/ml Curdlan and ROS formation was monitored for 90 min. ROS were quantified as area under the curve (AUC). N=2, n=6. Two-tailed unpaired Student's t-test. **b** The same experiment was repeated using mice with the same genotypes but on C57BL/6 background. N=2, n=6. Two-tailed unpaired Student's t-test. **c-e** Wt or 2b KO BMDC on C57BL/6 background were stimulated with 50 µg/ml dZym, 50 µg/ml Zym, MOI 10 HKCA, 200 µg/ml Curdlan or 500 ng/ml LPS. Secretion of TNF (**c**), IL-6 (**d**) and IL-1β (**e**) was quantified by ELISA. Two-tailed unpaired Student's t-test. N=2, n=6. **f** Wt or 2b KO BMDM

on BALB/c background were stimulated as described above and TNF in culture supernatants was analysed by ELISA. N=1, n=3. No statistics are displayed since no reaction of BMDMs to the most relevant ligands dZym and HKCA could be detected. A single experiment is depicted. **g** Prior to stimulation with either 50 µg/ml dZym or MOI 10 HKCA, wild type or SPPL2b-deficient BMDMs were incubated with 10 µg/ml anti-Dectin-1 blocking antibody (AB) for 30 min. Subsequently, ROS production was determined. dZym: N=3, n=9; HKCA: N=2, n=6. One-Way ANOVA with Tukey's *post hoc* test. p values from statistical testing for comparison of each condition to wild type control samples (from left to right) are: dZym: p<0.0001, p=0.2801, p=0.99; HKCA: p<0.0001, p=0.9983, p=0.1402. **h-j** Wild type or 2b KO BALB/c BMDC were pre-incubated with 10 µg/ml anti-Dectin-1 and subsequently treated with either 50 µg/ml dZym or MOI 10 HKCA. Levels of TNF (**h**), IL-6 (**i**) and IL-1β (**j**) in cell culture supernatants were assessed by ELISA. TNF, dZym: N=2, n=6; TNF, HKCA: N=3, n=8 (2b KO HKCA) or n=9 (rest); IL-6, dZym: N=3, n=9; IL-6 HKCA: N=2, n=6; IL-1β, dZym: N=3, n=9; IL-1β, HKCA: N=3, n=9. One-Way ANOVA with Tukey's *post hoc* test. p values from statistical testing for comparison of each condition to wild type control samples (from left to right) are: h, dZym: p=0.4881, p=0.9973, p>0.999; h, HKCA: p=0.9040, p=0.1387, p=0.3035. i, dZym: p=0.7558, p=0.991, p=0.9539; i, HKCA: p=0.9747, p=0.2621, p>0.999. j, dZym: p=0.0672, p=0.4725, p=0.8344; j, HKCA: p=0.1823, p=0.4396, p=0.7888. All bars depict Mean±SD. ns = not significant; * p≤0.05; ** p≤0.01; *** p≤0.001.

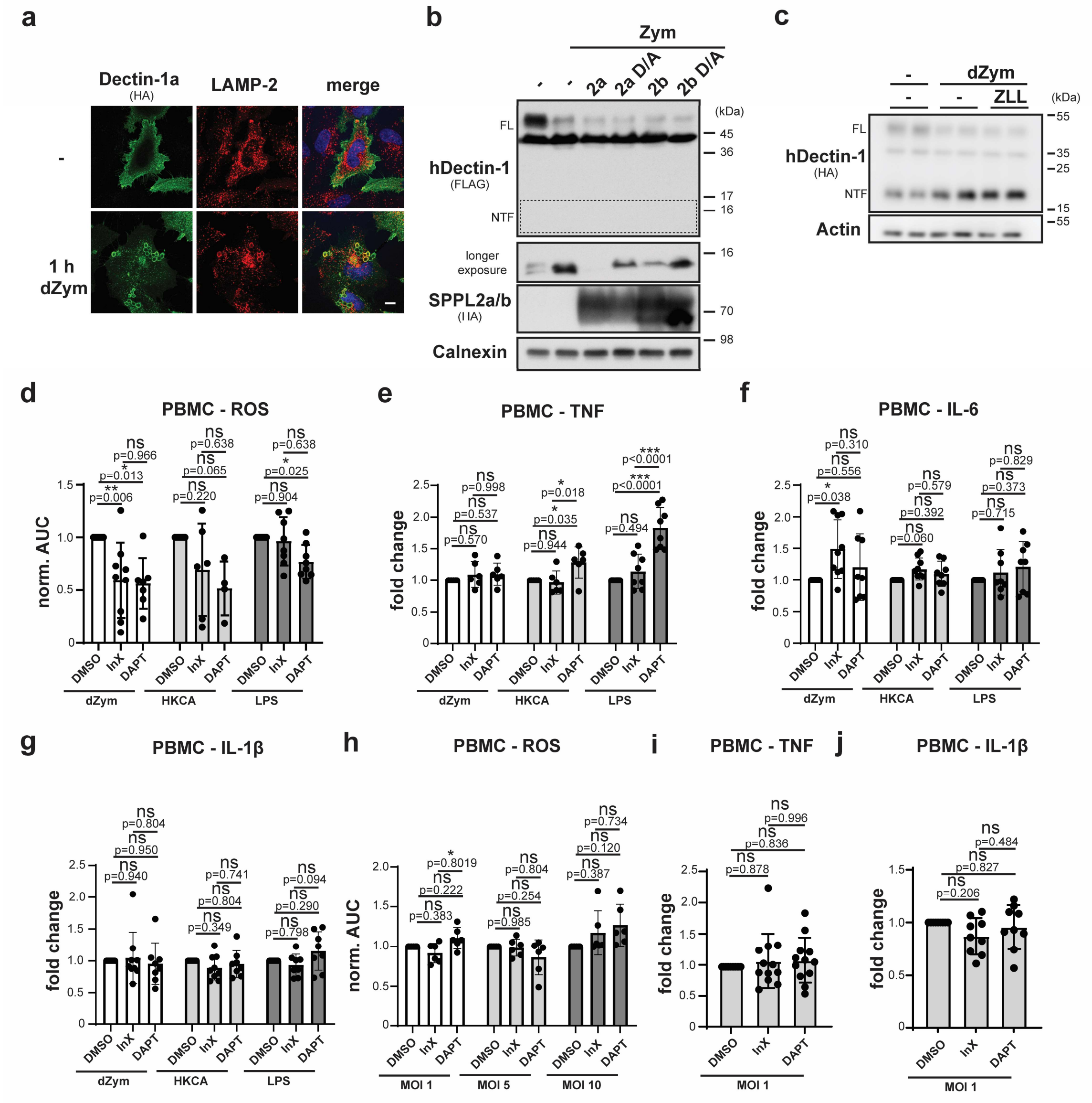

SUPPL. FIGURE 6: The role of SPPL2 proteases for processing of human Dectin-1a and anti-fungal responses in human PBMCs. a HeLa cells were transiently transfected with HA-hDectin-1a and either left untreated or stimulated with 50 µg/ml depleted Zymosan (dZym) for 1 h. Subsequently, cells were fixed using 4% PFA and analysed by immunofluorescence stainings using anti-HA for localization of Dectin-1a. Lysosomal compartments were visualised with LAMP-2. Scale bar, 10 µm. N=2, n=2. **b** T-Rex^{TM}-293 (HEK293) cells were transiently transfected with Flag-hDectin-1a-V5 either alone or together with HA-tagged active or inactive (D/A) human SPPL2a (2a) or SPPL2b (2b). Where indicated, cells were additionally stimulated with 100 µg/ml Zymosan (Zym) for 6 h. Membrane fractions were analysed by Western Blotting for proteolytic processing of Dectin-1. Throughout the Figure, full length (FL) hDectin-1a as well as the corresponding N-terminal fragment (NTF) are highlighted. N=4, n=4. **c** HEK cells transiently expressing HA-hDectin-1a were pre-incubated for 30 min with either DMSO or 40 µM ZLL and then stimulated with 50 µg/ml depleted Zymosan (dZym) for 6 h prior to Western Blot analysis. N=4, n=8. **d** Human PBMC were pretreated with 1 µM InX or 10 µM DAPT for 30 min and then stimulated with 50 µg/ml dZym, MOI 10 HKCA or 500 ng/ml LPS. ROS formation was measured using the luminometric L-012 probe and calculated as area under the curve (AUC). dZym: N=3, n=9; HKCA: DMSO and Inx: N=3, n=6, DAPT: N=2, n=4; LPS: N=3, n=8. Two-tailed unpaired Student's t-test. **e-g** PBMC were incubated with 1 µM InX or 10 µM DAPT prior to application of either 50 µg/ml dZym, MOI 10 HKCA or 500 ng/ml LPS. Amounts of secreted TNF (**e**), IL-6 (**f**) and IL1β (**g**) were determined by

ELISA. N=2/3, n=6-8. e, dZym and HKCA: N=2, n=6, LPS: N=3, n=8. f and g, dZym and HKCA: N=3, n=9 (DMSO and InX), N=3, n=8 (rest). **h** Same setup as described in d), but cells were stimulated with living *C. albicans* yeasts at MOIs of 1, 5 or 10, respectively. N=2, n=6. Two-tailed unpaired Student's t-test. **i,j** After incubation with either 1 μM InX or 10 μM DAPT for 30 min, human PBMC were stimulated with living *C. albicans* yeasts with a MOI of 1 for 24 h. Levels of TNF (**i**) and IL-1β (**j**) in the respective supernatants were quantified by ELISA. TNF: N=4, n=12; IL-1β: N=3, n=9. One-Way ANOVA with Tukey's *post hoc* test. All bars depict Mean±SD. ns = not significant; * $p \leq 0.05$; ** $p \leq 0.01$; *** $p \leq 0.001$.

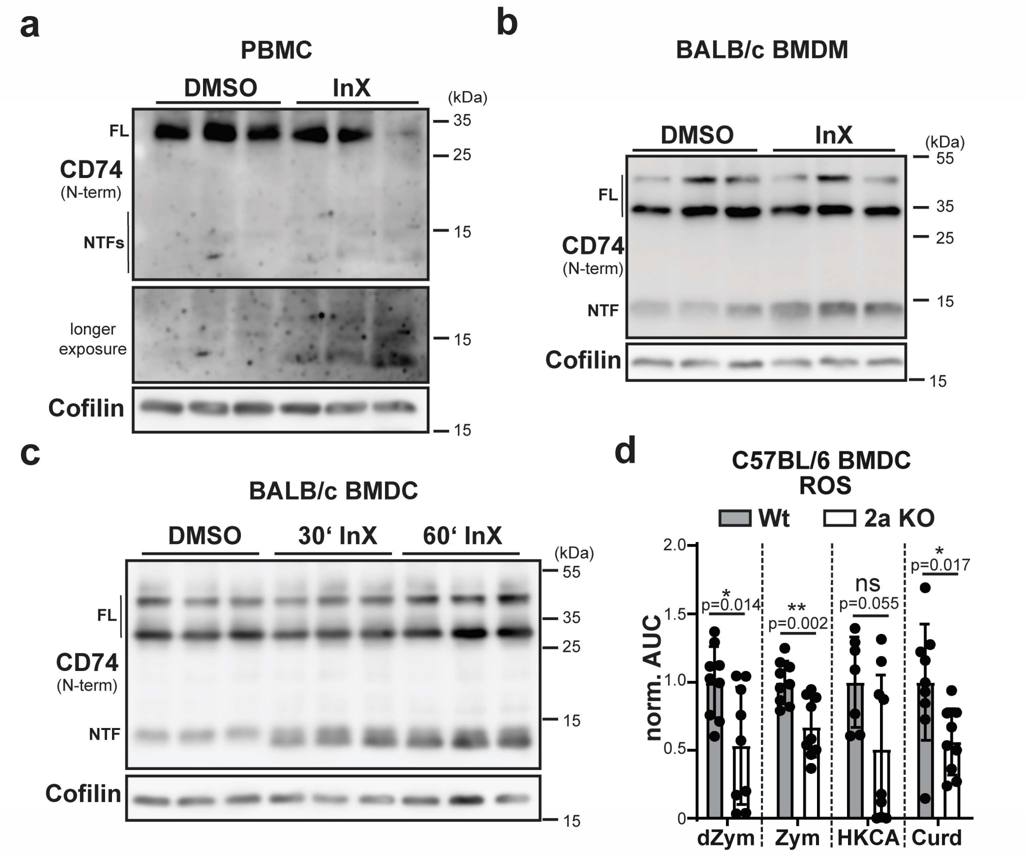

SUPPL. FIGURE 7: Inhibition of SPPL2a leads to rapid accumulation of CD74 N-terminal fragments and impairs ROS production. a Human PBMC were treated for 1 h with 1 µM InX and CD74 levels were analysed by Western blotting. Full length (FL) as well as the respective N-terminal fragments (NTFs) of CD74 are labelled throughout the Figure. N=1, n=3. **b** The same experiment was conducted employing wild type BALB/c BMDM instead of PBMC. N=2, n=6. **c** Wild type BALB/c BMDC were treated for 30 or 60 min with 1 µM InX. CD74 protein amounts were finally visualised by Western blotting. N=2, n=6. **d** Wild type or SPPL2a-deficient (2a KO) C57BL/6 BMDC were treated with either 50 µg/ml dZym, 50 µg/ml Zym, MOI 10 HKCA or 200 µg/ml Curdlan and ROS formation was measured using the luminescent L-012 probe. ROS were quantified as area under the curve (AUC). N=3, n=7 (HKCA) or n=9 (rest). Two-tailed unpaired Student's t-test. All bars depict Mean±SD. ns, not significant; * p≤0.05; ** p≤0.01.

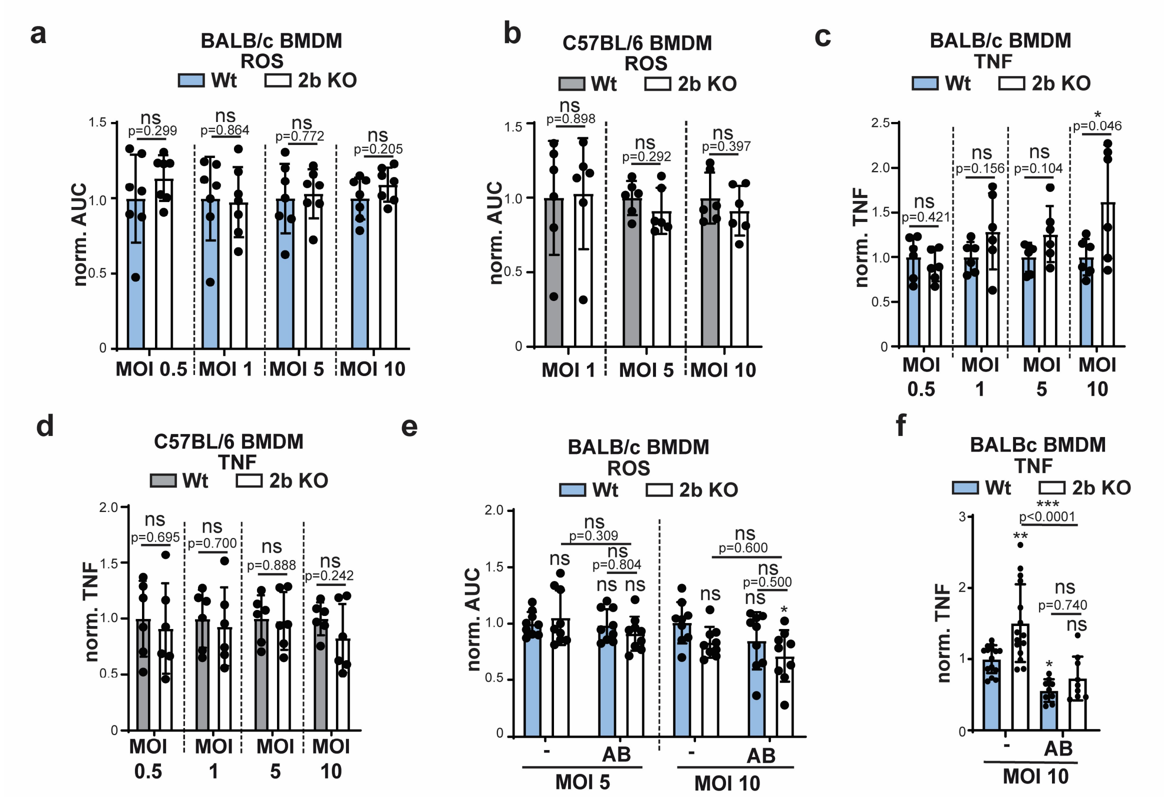

SUPPL. FIGURE 8: Effects of SPPL2b deficiency on ROS and cytokine production by macrophages co-cultured with *C. albicans.* **a** Either wild type (Wt) or *SPPL2b*$^{-/-}$ (2b KO) BALB/c BMDM were treated with living *C. albicans* yeast with the indicated MOIs and ROS formation was detected using the luminometric L-012 probe. ROS were quantified as area under the curve (AUC). N=2, n=7. Two-tailed unpaired Student's t-test. **b** The same experiment was repeated employing BMDM with the same genotypes but on C57BL/6 background. N=2, n=6. Two-tailed unpaired Student's t-test. **c,d** TNF levels were quantified by ELISA in the supernatants of wild type or SPPL2b-deficient BMDM on either BALB/c (**c**) or C57BL/6 (**d**) background upon stimulation of these cells with living *C. albicans* yeast employing the indicated MOIs. N=2, n=6 in both cases. Two-tailed unpaired Student's t-test. **e** BALB/c BMDM obtained from Wt or 2b KO mice were pre-treated with 10 µg/ml anti-Dectin-1 (AB) for 1 h and then stimulated with 50 µg/ml dZym or MOI 10 HKCA. ROS formation was detected using the luminometric L-012 probe for 90 min. The area under the curve (AUC) was calculated from the respective curves to quantify ROS formation. N=3, n=9. One-Way ANOVA with Tukey's *post hoc* test. p values from statistical testing for comparison of each condition to wild type control samples (from left to right) are: MOI 5: p=0.9065, p=0.9976, p=0.6976; MOI 10: p=0.2903, p=0.3716, p=0.0223. **f** Wt or 2b KO BALB/c BMDM were incubated with 10 µg/ml anti-Dectin-1 prior to treatment with living *C. albicans* yeast at a MOI of 10. TNF levels in the culture supernatants were measured by ELISA. N=5 (no AB)/3 (+ AB), n=15 (no AB)/9 (+ AB). One-Way ANOVA with Tukey's *post hoc* test. p values from statistical testing for comparison of each condition to wild type control samples (from left to right) are: p=0.0021, p=0.0266, p=0.2919. All bars depict Mean±SD. ns = not significant; * p≤0.05; ** p≤0.01; *** p≤0.001.

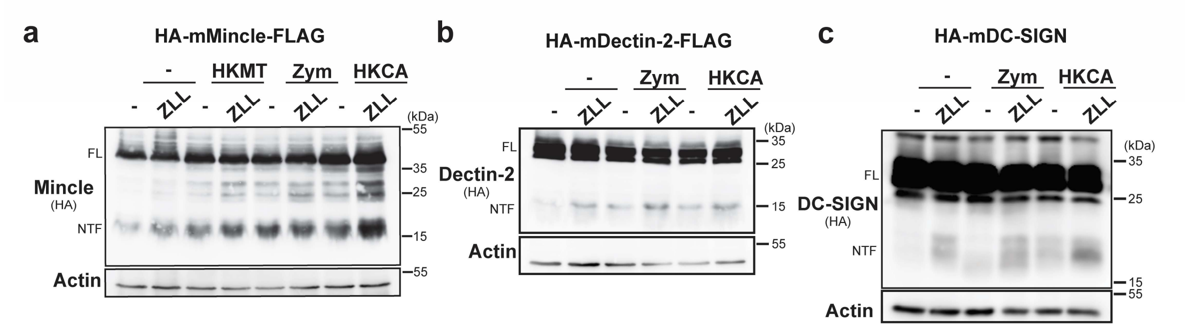

SUPPL. FIGURE 9: N-terminal fragments derived from the murine C-type lectin receptors Mincle, Dectin-2 and DC-SIGN are stabilised by inhibition of SPPL2 proteases. **a** J774.E cells stably transduced with HA-mMincle-FLAG were pre-treated for 30 min with either 40 µM ZLL or the respective volume of DMSO (-) as control. Afterwards, cells were incubated for 6 h with 250 µg/ml heat-killed *M. tuberculosis* (HKMT), 50 µg/ml Zymosan (Zym) or MOI 10 heat-killed *C. albicans* (HKCA) and analysed for processing of Mincle by Western Blotting. N=3, n=3. **b** SPPL2 proteases were inhibited in J774.E cells stably overexpressing HA-mDectin-2-FLAG by 40 µM ZLL starting 30 min prior to stimulation of the cells with either 50 µg/ml Zym or MOI 10 HKCA. Proteolysis of Dectin-2 was assessed by Western Blotting. N=3, n=3. **c** HEK cells transiently transfected with HA-mDC-SIGN were treated as described in b) and analysed for processing of DC-SIGN. Bands representing the full length forms (FL) of the respective receptors and the derived NTFs are labelled. N=3, n=3.

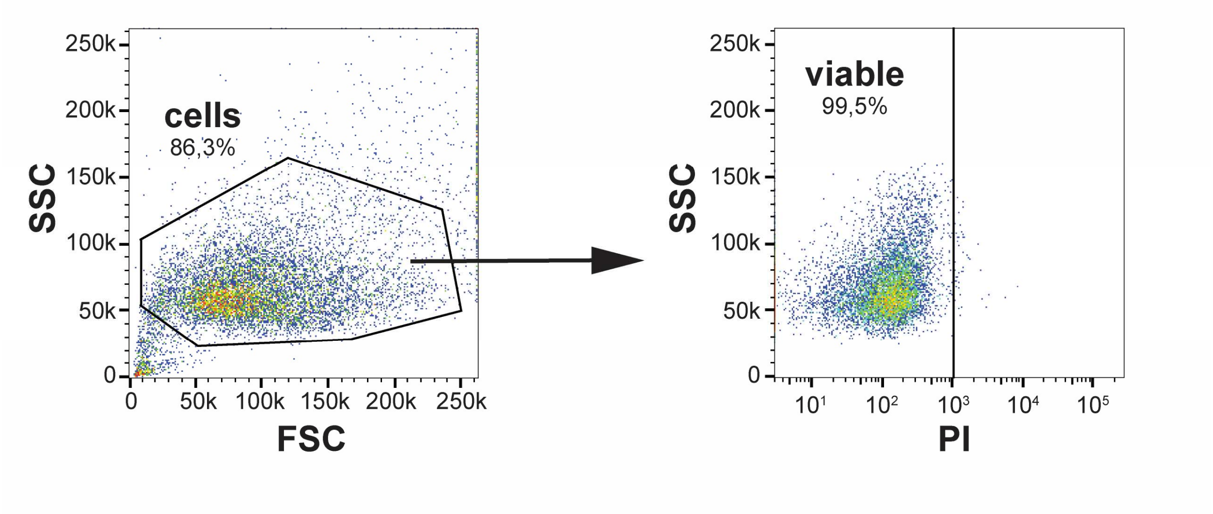

SUPPL. FIGURE. 10: Gating scheme for flow cytometric quantification of Dectin-1 surface levels in stably transfected HEK cells. After exclusion of debris from side scatter (SSC)/forward scatter (FSC) plots, cells were divided into propidium iodide (PI) positive dead cells and PI negative living cells. Finally, Dectin-1 PE staining intensity of the PI negative (viable) population was evaluated.

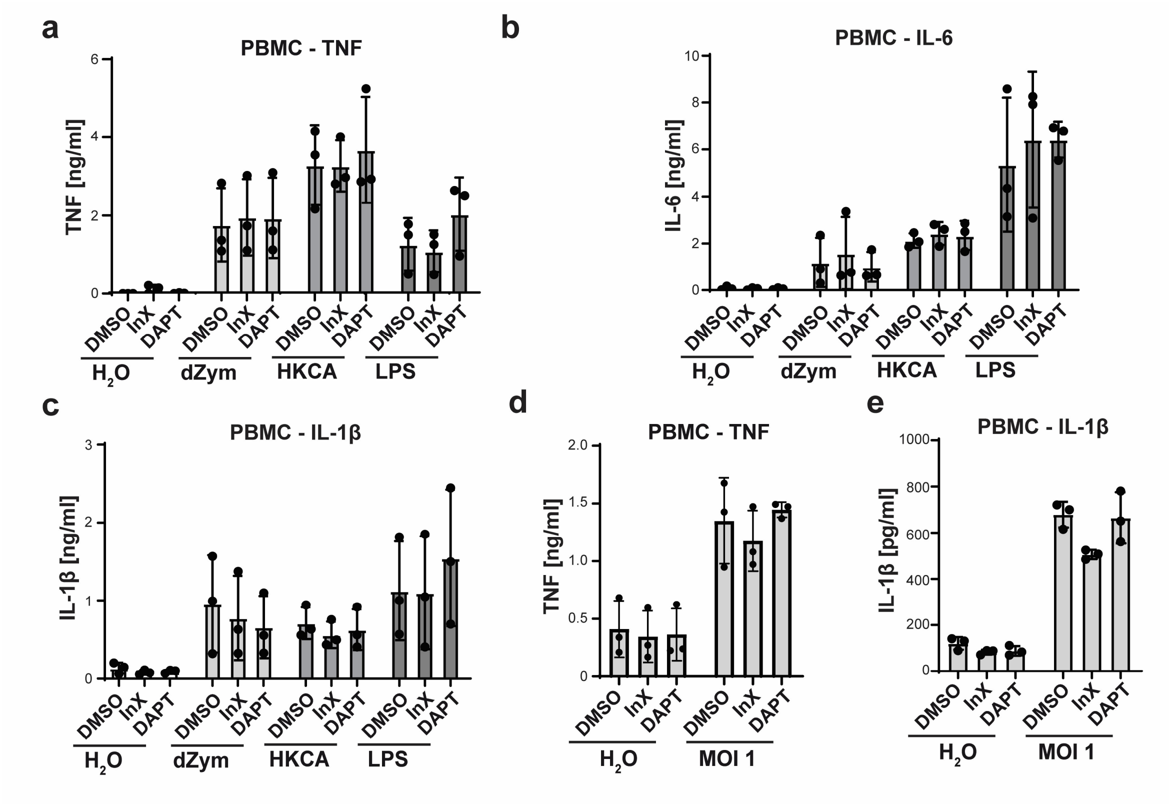

SUPPL. FIGURE 11: Representative cytokine concentration data for PBMC experiments. Cytokine concentrations are provided from a representative PBMC experiments (N=1, n=3 in all cases) including H_2O-treated control samples corresponding to the pooled datasets depicted in Suppl. Fig. 6e (**a**), Suppl. Fig. 6f (**b**), Suppl. Fig. 6g (**c**), Suppl. Fig. 6i (**d**) and Suppl. Fig 6j (**e**). All bars depict Mean values ± SD.

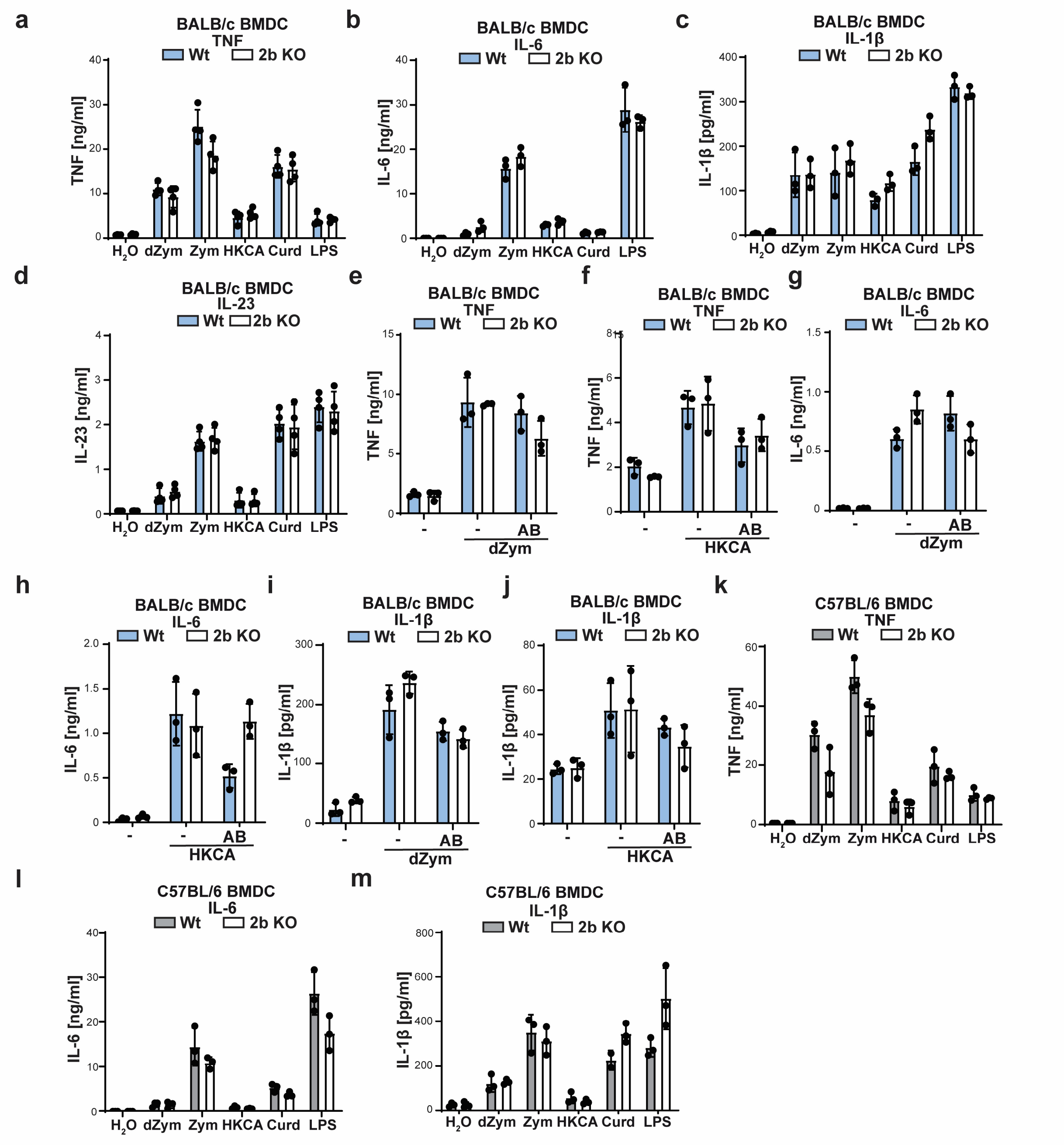

SUPPL. FIGURE 12: Representative cytokine concentration data for BMDC experiments employing artificial Dectin-1 ligands. Cytokine concentrations are depicted from representative BMDC experiments including H_2O-treated control samples corresponding to the pooled datasets shown in Fig. 5e (**a**), Fig. 5f (**b**), Fig. 5g (**c**), Fig. 5h (**d**), Suppl. Fig. 5h (**e**,f), Suppl. Fig. 5i (**g**,**h**), Suppl. Fig. 51 (**i**,**j**), Suppl. Fig. 5c (**k**), Suppl. Fig. 5d (**l**) and Suppl. Fig. 5e (**m**). For a,d N=1, n=4, for the rest N=1, n=3 (m: Curd 2b KO: n=2). All bars depict Mean values ± SD.

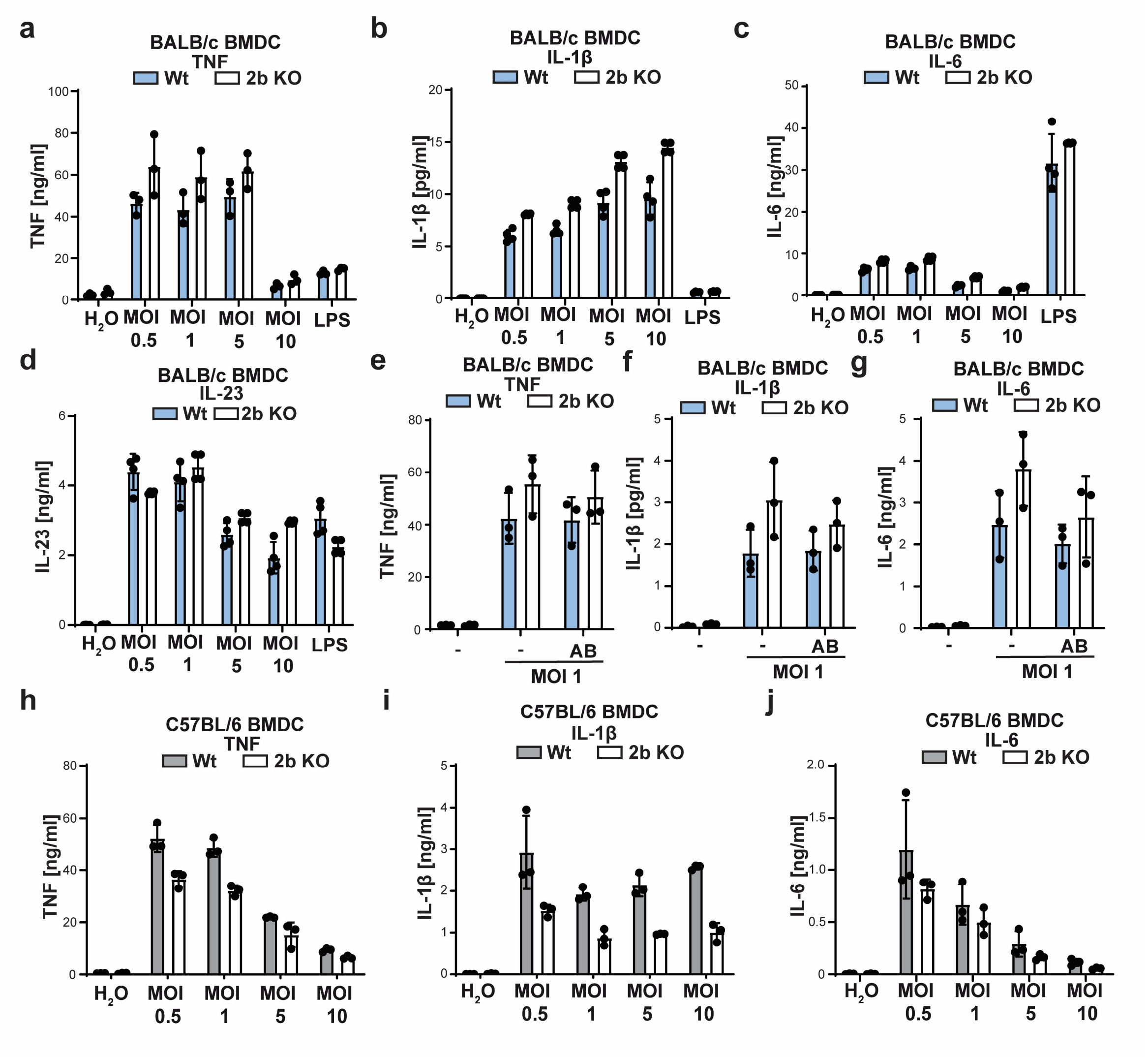

SUPPL. FIGURE 13: Representative cytokine concentration data for BMDC experiments involving treatment with living *C. albicans* yeasts. Cytokine concentrations are provided from representative BMDC experiments including H_2O-treated control samples corresponding to the pooled datasets depicted in Fig. 6e (**a**), Fig. 6f (**b**), Fig. 6g (**c**), Fig. 6h (**d**), Fig. 6m (**e**), Fig. 6n (**f**), Fig 6o (**g**), Fig 6i (**h**), Fig 6j (**i**) and Fig 6k (**j**). For a-d N=1, n=4, for the rest N=1, n=3. All bars depict Mean values ± SD.

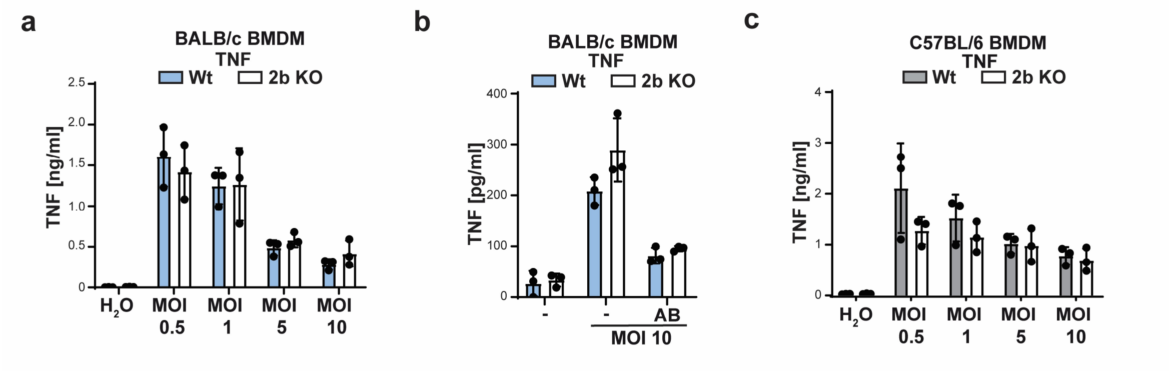

SUPPL. FIGURE 14: Representative cytokine concentration data for BMDM experiments. Cytokine concentrations are provided from representative BMDM experiments including H_2O-treated control samples corresponding to the pooled datasets shown in Suppl. Fig. 8c (a), Suppl. Fig. 8f (b) and Suppl. Fig 8d (c). In all cases N=1, n=3. All bars depict Mean values ± SD.
