# Supplementary material for: Phagosomal signalling of the C-type lectin receptor Dectin-1 is terminated by intramembrane proteolysis
Source: Nat Commun. 2022 Apr 6;13:1880. doi: 10.1038/s41467-022-29474-3 (PMC8987071; doi:10.1038/s41467-022-29474-3)
Supplement: Supplementary file 3 — Reporting summary [file 41467_2022_29474_MOESM3_ESM.pdf]

nature research

Corresponding author(s): Bernd Schröder

Last updated by author(s): 08.02.2022

Reporting Summary

Nature Research wishes to improve the reproducibility of the work that we publish. This form provides structure for consistency and transparency in reporting. For further information on Nature Research policies, see our Editorial Policies and the Editorial Policy Checklist.

Statistics

For all statistical analyses, confirm that the following items are present in the figure legend, table legend, main text, or Methods section.

n/a	Confirmed	
☐	☒	The exact sample size (*n*) for each experimental group/condition, given as a discrete number and unit of measurement
☐	☒	A statement on whether measurements were taken from distinct samples or whether the same sample was measured repeatedly
☐	☒	The statistical test(s) used AND whether they are one- or two-sided<br>*Only common tests should be described solely by name; describe more complex techniques in the Methods section.*
☒	☐	A description of all covariates tested
☐	☒	A description of any assumptions or corrections, such as tests of normality and adjustment for multiple comparisons
☐	☒	A full description of the statistical parameters including central tendency (e.g. means) or other basic estimates (e.g. regression coefficient) AND variation (e.g. standard deviation) or associated estimates of uncertainty (e.g. confidence intervals)
☐	☒	For null hypothesis testing, the test statistic (e.g. F, t, r) with confidence intervals, effect sizes, degrees of freedom and P value noted<br>*Give P values as exact values whenever suitable.*
☒	☐	For Bayesian analysis, information on the choice of priors and Markov chain Monte Carlo settings
☒	☐	For hierarchical and complex designs, identification of the appropriate level for tests and full reporting of outcomes
☒	☐	Estimates of effect sizes (e.g. Cohen's d, Pearson's r), indicating how they were calculated

Our web collection on statistics for biologists contains articles on many of the points above.

Software and code

Policy information about availability of computer code

Data collection	No software was used for data collection.
Data analysis	ImageJ (1.52a)<br>FlowJo (V10.6.0)<br>GraphPad Prism (8.4.0)<br>Excel 2016<br>GIMP (2.10.14)<br>Adobe Photoshop (CS6)

For manuscripts utilizing custom algorithms or software that are central to the research but not yet described in published literature, software must be made available to editors and reviewers. We strongly encourage code deposition in a community repository (e.g. GitHub). See the Nature Research guidelines for submitting code & software for further information.

Data

Policy information about availability of data

All manuscripts must include a data availability statement. This statement should provide the following information, where applicable:

- Accession codes, unique identifiers, or web links for publicly available datasets
- A list of figures that have associated raw data
- A description of any restrictions on data availability

The authors declare that all data supporting the findings of this study are available within the article and its Supplementary Information files. Source data are provided with this paper.

Field-specific reporting

Please select the one below that is the best fit for your research. If you are not sure, read the appropriate sections before making your selection.

- [x] Life sciences
- [ ] Behavioural & social sciences
- [ ] Ecological, evolutionary & environmental sciences

For a reference copy of the document with all sections, see nature.com/documents/nr-reporting-summary-flat.pdf

Life sciences study design

All studies must disclose on these points even when the disclosure is negative.

Sample size	No sample size calulation was performed. Instead, sample sizes were chosen based on established and accepted standards of the field. In general, experiments were repeated at least 3 times using independent samples. When differences were small, experiments were repeated with up to 15 mice to be sure to give a good estimation of normal variation within the experiment.
Data exclusions	Individual values were excluded from the following Figures due to high deviation from other data and are highlighted in the accompanying Source data file:<br>Fig. 6d: one sample from SPPL2b KO MOI 10 (reason: deviation more than 5-fold standard deviation)<br>Fig. 6n: data originating from a single knockout animal due to an in general impaired response of cells derived from this mouse to the applied ligands (more than 2-3 fold of standard deviation)<br>Fig. 6o: data from the same Knockout animal as in Firgure 6n were excluded for the same reasons mentioned above.<br>Suppl. Fig. 7h: One sample from Knockout BMDC treated with HKCA was excluded due to high deviation (approx. 5-fold standard deviation)<br>Suppl. Fig. 7e: Two values from wild type BMDC treated with Curdlan were excluded due to high deviation (more than 5-fold standard deviation)
Replication	All experiments were replicated as indicated in the respective figure legends.
Randomization	For mouse experiments, animals were grouped to the experimental sets to obtain age- and sex-matched cohorts based on the assigned genotypes without further selection. For PBMC experiments, blood samples were provided by the German Red Cross Blood Donation Service North-East, Dresden or the Transfusion Medicine of the University Hospital Schleswig-Holstein (UKSH) in Kiel, which randomly selected healthy volunteers.
Blinding	Blinding was not employed since most of the readouts were based on machine readouts (ROS, ELISA, FACS) and thereby could be only minimally influenced by the experimentator. Western Blot results were also quantified to minimise subjective judgements. Based on limited staff and special restrictions in the context of working with viable C. albicans yeasts, the person performing the experiment usually had to be the same also evaluating it.

Reporting for specific materials, systems and methods

We require information from authors about some types of materials, experimental systems and methods used in many studies. Here, indicate whether each material, system or method listed is relevant to your study. If you are not sure if a list item applies to your research, read the appropriate section before selecting a response.

Materials & experimental systems

n/a	Involved in the study	
[ ]	[x]	Antibodies
[ ]	[x]	Eukaryotic cell lines
[x]	[ ]	Palaeontology and archaeology
[ ]	[x]	Animals and other organisms
[ ]	[x]	Human research participants
[x]	[ ]	Clinical data
[x]	[ ]	Dual use research of concern

Methods

n/a	Involved in the study	
[x]	[ ]	ChIP-seq
[ ]	[x]	Flow cytometry
[x]	[ ]	MRI-based neuroimaging

Antibodies

Antibodies used	anti-Dectin-1-PE (Biolegend, clone RH1, Cat. no. 144303); FACS: 1/200<br>Anti-Dectin-1 N-terminus (custom-made by Pineda Antikörper Service, validated in Gradtke et al., 2019 and within the manuscript); WB: 1/2000<br>Anti-Dectin-1 (for blocking, clone 2A11, BioRad, Cat. no. MCA2289); blocking experiments: 10 µg/ml<br>anti-mSPPL2a (custom-made by Pineda Antikörper Service, described in Behnke et al., 2011); WB: 1/1000<br>anti-mSPPL2b (custom-made by Pineda Antikörper Service, described Schneppenheim et al., 2014); WB: 1/200<br>anti-hSPPL2a (custom-made by Pineda Antikörper Service, described in Schneppenheim et al., 2014); WB: 1/1000<br>anti-hSPPL2b (custom-made by Pineda Antikörper Service, described in Mentrup et al., 2019); WB: 1/1000<br>anti-hCD74 (PIN.1, StressMarq Biosciences, Cat.no. SMC-116D); WB: 1/1000<br>anti-mCD74 (In-1, BD Biosciences, Cat. no. 555317 RRID:AB_395727); WB: 1/10000<br>anti-Syk (Cell Signaling, clone D3Z1E, Cat. no. 13198S); WB:1/1000, IP: 1/500<br>anti-GFP (Cell Signaling, clone D5.1, Cat. no. 2956T); WB: 1/2000<br>anti-Actin (Sigma, Cat. no. A2066-100UL); WB: 1/4000<br>anti-Cofilin (Cell Signaling, clone D3F9, Cat. no. 5175S); WB: 1/2000<br>anti-EEF2 (Abcam, Cat. no. ab33523); WB: 1/2000<br>anti-alphaTubulin (Cell Signaling, Cat. no. 2144S); WB: 1/2000<br>anti-phospho-ERK1/2 (T202/Y204) (Cell Signaling, clone D13.14.4E, Cat. no. 4370S); WB: 1/1000<br>anti-ERK1/2 (Cell Signaling, clone 137F5, Cat. no. 4695S); WB: 1/2000<br>anti-phosphorylated Tyrosine (Santa Cruz Biotechnology, clone PY99, Cat. no. sc-7020); IP: 1/200<br>anti-HA (Roche, clone 3F10, Cat. no. 11867431001); WB: 1/2000, IF:1/200, IP: 1/500<br>anti-myc (Cell Signaling, clone 9B11, Cat. no. 2276S); WB: 1/2000, IF: 1/200<br>anti-LAMP2 (clone 2D5, Radons et al., 1992); IF: 1/300<br>anti-FLAG (Sigma, clone M2, F1804-50UG); WB: 1/1000<br>anti-Calnexin (Cell Signaling, Cat. no. 2679T); IF: 1/200<br>anti-Calnexin (Enzo Life Sciences, ADI-SPA-860-F); WB: 1/2000<br>Goat anti-Rat IgG (H+L) Cross-Adsorbed Secondary Antibody, Alexa Fluor 488 (Thermo Fisher, Cat. no. A-11006); IF: 1/500<br>Goat anti-Mouse IgG (H+L) Highly Cross-Adsorbed Secondary Antibody, Alexa Fluor 594 (Thermo Fisher, Cat. no. A-11032); IF: 1/500<br>Goat anti-Rabbit IgG (H+L) Highly Cross-Adsorbed Secondary Antibody, Alexa Fluor 594 (Thermo Fisher, Cat. no. A-11037); IF: 1/500<br>Goat IgG anti-Rat IgG (H+L)-HRPO, MinX Hu,Bo,Ho,Rb (Dianova, Cat. no. 112-035-143); WB: 1/20000<br>Goat IgG anti-Mouse IgG (H+L)-HRPO, MinX Hu,Bo,Ho,Rt,Rb (Dianova, Cat. no. 115-035-166); WB: 1/20000<br>Goat IgG anti-Rabbit IgG (H+L)-HRPO, MinX Hu,Ms,Rt (Dianova, Cat. no. 111-035-144); WB: 1/20000
Validation	anti-Dectin-1-PE: validated for FACS by company by staining of mouse peritoneal cells with antibody or respective isotype control<br>anti-Dectin-1 N-terminus: custom-made, validated for Western Blotting within the manuscript using a knockout control. Use for IF demonstrated in Gradtke et al., 2020. Only detects Dectin-1 transfected HeLa cells.<br>anti-Dectin-1 (blocking): Validated for blocking binding of ligands to Dectin-1 in Herre et al., 2004 and for further applications in Lin et al., 2010.<br>anti-mSPPL2a: custom-made, validated for Western Blot detection of murine SPPL2a in Behnke et al., 2011, Schneppenheim et al., 2014 and several other publications from our laboratory using knockout controls<br>anti-mSPPL2b: custom-made, validated for Western Blot detection of murine SPPL2b in Schneppenheim et al., 2014 and within the underlying manuscript using knockout controls<br>anti-hSPPL2a: knockout validated in Schneppenheim et al., 2014<br>anti-hSPPL2b: Validated by siRNA within the paper (Suppl. Fig. 3g)<br>anti-mCD74: Validated for detection of endogenous levels of CD74 and its corresponding NTF in Schneppenheim et al., 2013. Knockout validated in Bikoff et al., 1993.<br>anti-hCD74: validated by company for Western Blotting using human N87 cell lysates. Additionally validated in Roche et al., 1991 and Kong et al., 2018.<br>anti-Syk: validated by company for IP (human Syk) and Western blotting (murine) by appearance of a band at the corresponding size<br>anti-GFP: validated by company for detection of GFP in transiently GFP-transfected cells vs untransfected cells<br>anti-Actin: validated for Western Blotting of human and murine cells by company based on a band at corresponding size<br>anti-Cofilin: validated for Western Blot analysis of Cofilin protein levels in human and murine cell lines based on a band at the corresponding size<br>anti-EEF2: validated by company for Western Blotting of lysates of human and murine cell lines as well as murine tissue samples<br>anti-alphaTubulin: validated by company for Western Blotting using whole cell lysates of murine and human origin. Also validated using recombinant protein.<br>anti-phospho-ERK1/2: validated for use in Western Blot applications by company for human and murine cell lines based on a specific double band only occuring after stimulation of cells with TPA or other activators. Band can be blocked by application of an ERK1/2 inhibitor.<br>anti-ERK1/2: validated by company for Western Blotting in human and murine samples. Detecting a double band at the expected size. Specificity of the antibody has been validated by siRNA in HEK293T cells.<br>anti-phospho-Tyrosine: Validated by company for Western Blot application using lysates of unstimulated or EGF-treated A-431 cells (human). Specifically precipitates overexpressed Dectin-1 only upon stimulation with Zymosan known to induce tyrosine-phosphorylation of the receptor.<br>anti-HA: validated for Western Blot and IF detection of HA-tagged proteins in several publications from our laboratory, e.g. Mentrup et al., 2019.<br>anti-LAMP2: Specifically detects human LAMP2 in immunofluorescence analysis as indicated by clear vesicular/lysosomal staining. Was employed before several times in studies from our laboratory (e.g. Mentrup et al., 2019).

anti-myc: Specificity in Western Blot applications demonstrated by specific detection of myc-tagged proteins in HEK cells. Same for IF in HeLa cells (both shown within the manuscript). Also demonstrated by company.
anti-FLAG: validated for detection of FLAG-tagged proteins in Western Blot and Immunofluorescence applications (References for different applications listed at supplier's website: https://www.sigmaaldrich.com/catalog/product/sigma/f1804?lang=de&region=DE
anti-Calnexin (Enzo): Validated by company for use in Western Blotting for human cell lines.
anti-Calnexin (Cell Signaling): Validated by company for use in IF for human cell lines.
Goat anti-Rat IgG (H+L) Cross-Adsorbed Secondary Antibody, Alexa Fluor 488: Validated for use in IF by company
Goat anti-Mouse IgG (H+L) Highly Cross-Adsorbed Secondary Antibody, Alexa Fluor 594: Validated for use in IF by company
Goat anti-Rabbit IgG (H+L) Highly Cross-Adsorbed Secondary Antibody, Alexa Fluor 594: Validated for use in IF by company
Goat IgG anti-Rat IgG (H+L)-HRPO, MinX Hu,Bo,Ho,Rb (Dianova, Cat. no. 112-035-143): Validated for use in Western Blotting by company
Goat IgG anti-Mouse IgG (H+L)-HRPO, MinX Hu,Bo,Ho,Rt,Rb (Dianova, Cat. no. 115-035-166): Validated for use in Western Blotting by company
Goat IgG anti-Rabbit IgG (H+L)-HRPO, MinX Hu,Ms,Rt (Dianova, Cat. no. 111-035-144): Validated for use in Western Blotting by company

Eukaryotic cell lines

Policy information about cell lines

Cell line source(s)	HeLa cells: German Collection of Microorganisms and Cell Cultures<br>HEK293T cells: German Collection of Microorganisms and Cell Cultures<br>J774.E cells: originally generated by subcloning from J774 cells by Philip Stahl (Diment et al., 1987) and provided to us by Albert Haas, Bonn, Germany. Commercially not available, no information of the original source of cells used for subcloning is not available.<br>MEF (Wt and SPPL2a/b dKO): generated in the laboratory. Described in Mentrup et al., 2019.<br>FlipIn 293 cells: Invitrogen<br>T-REX 293 cells: Invitrogen<br>HEK 293 SPPL2A knockout cells: Cells were based on T-REX 293 cells described above, generation is presented in detail in Spitz et al., 2020
Authentication	Cell lines were not authenthicated within the laboratory.
Mycoplasma contamination	Cell lines were not tested for mycoplasma contamination
Commonly misidentified lines (See ICLAC register)	No commonly misidentified cell lines were used.

Animals and other organisms

Policy information about studies involving animals; ARRIVE guidelines recommended for reporting animal research

Laboratory animals	mouse: C57BL6/NCrl: both male and female, 10-20 weeks old<br>mouse: CB-3110056O03RikGt(pU-21T)160Imeg on Balb/cAnNCrland C57BL/6NCrl background: male and female, 10-20 weeks old<br>mouse: Sppl2a Knockout on C57BL/6 background: male and female, 10-20 weeks old, originally described in Schneppenheim et al., 2013
Wild animals	Study did not involve wild animals.
Field-collected samples	Study did not involve field-collected samples
Ethics oversight	Experiments were approved by the Ministerium für Energiewende, Landwirtschaft, Umwelt und ländliche Räume of Schleswig-Holstein (V 242.7224.121-3) and the Landesdirektion Sachsen (TV A 12/2018, DD24.1-5131/450/12).

Note that full information on the approval of the study protocol must also be provided in the manuscript.

Human research participants

Policy information about studies involving human research participants

Population characteristics	Leukocyte concentrates were obtained from healthy adult blood donors. Age and sex of the corresponding donors was not specified.
Recruitment	PBMCs were isolated from blood samples obtained with written informed consent from healthy donors by the German Red Cross Blood Donation Service North-East, Dresden or from the Transfusion Medicine of the University Hospital Schleswig-Holstein (UKSH) in Kiel. Blood samples were randomly provided by the above-mentioned institutions without any influence from our group on selection of individuals.
Ethics oversight	The study was approved by the local institutional review board of the Medical Faculty Carl Gustav Carus, TU Dresden, Germany (EK138042014) and the institutional ethic committee of the CAU Kiel (D517/15).

Note that full information on the approval of the study protocol must also be provided in the manuscript.

Flow Cytometry

Plots

Confirm that:

- [x] The axis labels state the marker and fluorochrome used (e.g. CD4-FITC).
- [x] The axis scales are clearly visible. Include numbers along axes only for bottom left plot of group (a 'group' is an analysis of identical markers).
- [x] All plots are contour plots with outliers or pseudocolor plots.
- [x] A numerical value for number of cells or percentage (with statistics) is provided.

Methodology

Sample preparation	For staining of HEK cells stably overexpressing either HA-mDectin-1a-FLAG or HA-mDectin-1b-FLAG, cells were detached using Accutase, washed once with FACS buffer (2% FCS, 2 mM EDTA in PBS). For staining, cells were resuspended in 100 µl FACS buffer containing Dectin-1-PE (RH1, Biolegend) antibodies (diluted 1/200) and stained for 1 h on ice. Afterwards, cells were spun down and resuspended in 100 µl FACS buffer containing 250 ng/ml propidium iodide (Invitrogen) prior to a final washing step with FACS buffer. Finally, cells were washed once, resuspended in 200 µl FACS buffer and analyzed by flow cytometry using a FACS Canto II. Data processing was performed using FlowJo software (BD Biosciences). In the respective histograms, PE signals of PI negative cells are depicted.
Instrument	FACS Canto II and BD LSR II Flow Cytometry Cell Analyzer (both from Becton Dickinson)
Software	FlowJo 10.6.0
Cell population abundance	Dectin-1 positive and PI-negative samples typically made up to 95% of the analysed cell populations as jugded by comparisons with a corresponding isotype control antibody.
Gating strategy	Cell population was divided in PI positive (dead) and negative (living) cells which were analysed for their Mean PE-staining intensity.

- [x] Tick this box to confirm that a figure exemplifying the gating strategy is provided in the Supplementary Information.
